# Prevalence of intestinal parasitic infection and associated factors among pregnant women attending antenatal care at public health facilities in Lalo Kile district, Oromia, Western Ethiopia

**DOI:** 10.1186/s13104-019-4781-3

**Published:** 2019-11-08

**Authors:** Dejene Abraham Yesuf, Lensa Tesfaye Abdissa, Emiru Adeba Gerbi, Edosa Kifle Tola

**Affiliations:** 1 Kellam Wollega, Lalo Kile district, Oromia, Ethiopia; 2 Department of Medical Laboratory Science, Institute of Health Science, Wollega University, Nekemte, Ethiopia; 3 Department of Public Health, Institute of Health Science, Wollega University, Nekemte, Ethiopia

**Keywords:** Prevalence, Intestinal parasitic infection, Pregnant women, Lalo Kile district

## Abstract

**Objective:**

This study aimed to assess the prevalence of intestinal parasitic infection and associated factors among pregnant women attending antenatal care at public health facilities in Lalo Kile district, Oromia, Western Ethiopia.

**Results:**

Out of the 315 respondents, the mean age was 24.7 ± 2.54 years ranging between 15 and 44 years. The majority of the respondents were Oromo in ethnicity (90.2%) and protestant in religion (83.2%) and almost all (99%) of the study participants were married. Two hundred twenty-six (72%) of pregnant women were attended at least primary school and two hundred forty-six (78.1%) of the respondents were from farmer households. About half of the households (51.7%) had a monthly income of greater than 35 USD. The overall prevalence of intestinal parasitic infection was 138 (43.8%) with the predominance of hookworm (33.7%) followed by *Ascaris lumbricoides* (7.3%). Being a farmer [AOR, 95% CI 7.3 (1.46, 37.00), P = 0.03], walking barefooted [AOR, 95% CI 6.13 (1.98, 19.00), P = 0.002] and absence of proper handwashing after latrine [AOR, 95% CI 5.36 (1.78, 16.00), P = 0.003] were significantly associated with occurrence of the intestinal parasitic infection.

## Introduction

Intestinal parasitic infection (IPI) is a condition in which a parasite infects the gastrointestinal tract of humans. Parasitic infections, caused by intestinal helminths and protozoan parasites, are among the most prevalent infections in humans in developing countries. The difference between the two occurs in their cell structure. Protozoan parasites are single-celled while helminths are multi-cellular worms [1–3]. According to world health organization (WHO) estimates, soil-transmitted helminths (STH) affects 1.5 billion people worldwide (24% of the world population) with the greatest numbers occurring in sub-Saharan Africa [1, 2].

Intestinal parasitic infection is very common in Ethiopia and the magnitude of infection varies from place to place [4–8]. Intestinal parasitic infections account for the second most predominant causes of outpatient morbidity in the country [9]. High prevalence of parasitic infection in Ethiopia was due to the unsafe and inadequate provision of water, unhygienic living conditions, the absence of proper utilization of latrine and habit of walking with a barefoot [5, 10].

Tens of millions of pregnant women as one of the segments of the community are affected by parasitic infections which directly or indirectly lead to a spectrum of adverse maternal and fetal/placental effects [11]. Intestinal helminths are among the most common and widespread of human infections contributing to poor nutritional status, anemia and impaired growth [12].

The major IPI of global public health concern is the protozoan species like *E. histolytica* and *G. lamblia* and helminthic species like *A. lumbricoides*, *T. trichuria*, hookworm (*A. duodenale* and *N. americanus*), *E. vermicularis*, *Taenia* species and *S. mansoni* [2, 13].

IPI is associated with poverty conditions such as reduced access to safe drinking water, poor environmental sanitation and personal hygiene, inadequate access to health care, poor nutritional status, prevailing climatic and environmental conditions [14, 15].

Pregnancy is a physiological state which is often associated with changes in disease susceptibility. The change in immunity during pregnancy induces tolerance to fetal implantation and also associated with decreased immunity to various infections such as IPI. So, IPI is a double burden for pregnant women in affecting the health of both pregnant women and their offspring [9, 15]. IPI is a major public health problem in Ethiopia with the varied prevalence from place to place, but data is limited in the western part of the region. Therefore, the present study aims to fill this gap in the study area.

## Hypothesis (research questions)

What will be the prevalence of intestinal parasitosis in the study area? Which of the identified species becomes the most dominant? Which of the associated factors is predisposing for the occurrence of the intestinal parasitosis? These and others need answers.

## Main text

### Methods

A cross-sectional study was conducted from April 1 to May 15, 2019, in public health facilities of Lalo Kile district: Lalo Kile, Wabera, Hadere Haro and Serba health centers. Lalo Kile is located 555 km from the capital city of Ethiopia, Addis Ababa in the western and 103 km from Dambi Dollo in Eastern direction. The required sample size (n) was determined using single population proportion formula with the assumptions: estimated prevalence (P) of IPI (24.7%) [18], 95% C.I (1.96), 5% margin of error (d) and adding 10% contingency:

$${\text{n}} = \frac{{({\text{Z}}\alpha /2)^{2} \cdot {\text{p}}(1 - {\text{p}})}}{{{\text{d}}^{2} }} = 315$$

A systematic random sampling technique was employed to select the study subjects from ANC during the survey period. According to the health centers service delivery report, on average, 770 pregnant mothers have enrolled in ANC. A sampling interval of 2 was used to select participants after sample size was allocated proportionally to all HCs. Of the first two pregnant mothers, one was selected by the lottery method, eventually, every 2nd; the participant was selected for enrolment in the study.

Ethical clearance was obtained from the Research Ethics Review Committee (RERC) of Wollega University, Institute of Health Sciences, Department of Public Health. A formal letter of cooperation was written to the respective administrator of all HCs. Informed written consent was obtained from each study participant. Confidentiality was maintained and the unauthorized person did not access to the data collection and processing area. All identified parasites were treated according to the guidelines of the National helminth Control Program of Ethiopia [26].

For the sake of data quality, pre-testing was performed and also training was given to interviewers before data collection and periodic supervision was done by the principal investigator to check consistency and completeness of the data.

Using structured questionnaires, data such as socio-demographic, hygiene and environmental factors, and nutritional and dietary-related factors were collected by direct interview and reviewing of respondent’s medical records. A stool sample was collected and processed by direct wet mount and formalin-ether concentration techniques to identify intestinal parasites.

All data were cleaned, edited and checked for completeness and analyzed by using SPSS version 20 and displayed by tables. Descriptive statistics were employed to depict numbers and frequencies. Furthermore, odds ratio, 95% confidence interval, and P value were computed using a logistic regression model to assess the associations between dependent and independent variables. Variables having a P-value less than 0.05 with 95% confidence interval were declared as statistically significant.

### Results

Out of the 315 respondents, 132 (41.9%) of them fall within the 20–24 age range The mean age of the respondents was 24.7 ± 2.54 years ranging between 15 and 44 years. Nearly half, 132 (41.9%) of pregnant women were ranged between the ages of 20–24. The majority of the respondents were Oromo in ethnicity (90.2%) and protestant (83.2%) in religion. Almost all (99%) of the study participants were married. Two hundred twenty-six (72%) of pregnant women were attended at least primary school and two hundred forty-six (78.1%) of the respondents were from farmer households. More than half of the households (51.7%) had a monthly income of greater than 35 USD (Table 1).

**Table 1:** Socio-demographic characteristics of pregnant mothers on antenatal care visit in Lalo Kile district health facilities from April 1 to May 15, 2019 (n = 315)

Variables	N (%)
Age	
15–19	47 (14.9)
20–24	132 (41.9)
25–29	81 (25.7)
30–34	32 (10.2)
35–39	19 (6)
40–44	4 (1.3)
Ethnicity	
Oromo	284 (90.2)
Amhara	30 (9.5)
Gurage	1 (0.3)
Religion	
Protestant	262 (83.2)
Orthodox	41 (13)
Muslim	12 (3.8)
Marital status	
Married	312 (99)
Divorced	2 (0.6)
Widowed	1 (0.4)
Educational level	
No formal education	89 (28.3)
Primary school	128 (40.6)
Secondary school	72 (22.9)
College and above	26 (8.3)
Residence	
Urban	71 (22.5)
Rural	244 (77.5)
Occupation	
Government employee	69 (21.9)
Farmer	246 (78.1)
Average monthly income of the family^a^	
< 17.5	22 (7)
17.5–35	130 (41.3)
> 35	163 (51.7)
Family size	
≤ 2	70 (22.2)
≥ 3	245 (77.8)

^a^Average monthly income in USD (for year 2019)

Intestinal parasitic infection was observed in 138 pregnant women with an overall prevalence of 43.8%. In this study, five species of intestinal parasites were identified with a predominance of hookworm, detected in 106 (33.7%) of pregnant women followed by *Ascaris lumbricoides* with the prevalence of 23 (7.3%) (Table 2).

**Table 2:** Prevalence of intestinal parasites among pregnant mothers on ANC visits in Lalo Kile district health facilities from April 1 to May 15, 2019 (n = 315)

Intestinal parasites	N (%)
Hook worm	106 (33.7)
*Ascaris lumbricoides*	23 (7.3)
*Trichuris trichuria*	5 (1.6)
*Giardia lamblia*	3 (0.9)
*Strongyloides stercoralis*	1 (0.3)
Total	138 (43.8)

According to the binary logistic regression analysis, factors like attending only primary school, rural dwellers, average income of 17.5–35 USD, family size of ≥ 3 and washing their hands with water only before food preparation were considered to be clinically significant with P-value < 0.05 for the occurrence of intestinal parasitosis; however, variables like habit of eating unwashed fruits and vegetables, lack of water treatment, unavailability of latrine, and lack of access to health education was not significantly associated with IPI (P > 0.05) (Table 3).

**Table 3:** Logistic regression analysis of determinants associated with IPI among pregnant mothers on ANC visit at health facilities in Lalo Kile district from April 1 to May 15, 2019 (n = 315)

Variables	Intestinal parasites		COR (95% CI)^a^	P-value	AOR (95%CI)^b^	P-value	
	Present, n (%)	Absent, n (%)					
Educational status							
Primary school	61 (47.6)	67 (52.4)	3.8 (1.4, 10.76)	0.01	0.3 (0.02, 4.2)	0.38	
Secondary school	27 (37.5)	45 (62.5)	2.5 (0.8, 7.46)	0.09	0.68 (0.05, 8)	0.75	
College and above	5 (19.2)	21 (80.8)	1		1		
Place of residence							
Urban	16 (22.5)	55 (77.5)	1		1		
Rural	122 (50)	122 (50)	3.4 (1.8, 6.3)	0.001	0.34 (0.05, 2.1)	0.25	
Occupation							
Government employee	20 (28.9)	49 (71.1)	1		1		
Farmer	118 (47.9)	128 (52.1)	2.25 (1.2, 4)	0.006^*^	7.3 (1.46,37)	0.03^*^	
Average monthly income^c^							
< 17.5	11 (50)	11 (50)	1.58 (0.6, 3.87)	0.311			
17.5–35	64 (49.2)	66 (50.8)	1.53 (0.9,2.45)	0.07			
> 35	63 (38.7)	100 (61.3)	1				
Family size							
≤ 2	24 (34.3)	46 (65.7)	1				
≥ 3	114 (46.5)	131 (53.5)	1.7 (0.9, 2.9)	0.07			
Habit of eating unwashed fruits and vegetables							
Yes	60 (38.5)	96 (61.5)	0.67 (0.42,1)	0.10			
No	62 (48)	67 (52)	1				
Water treatment before drinking							
Yes	24 (40)	36 (60)	1				
No	114 (44.7)	141 (55.3)	1.2 (0.7, 2.15)	0.50			
Walking bare footed							
Yes	91 (66)	47 (34)	5.3 (3.3, 8.7)	0.001*	6.13 (1.98, 19)		0.002*
No	47 (26.5)	130 (73.5)	1		1		
Hand washing before food preparation (with)							
Soap and water	40 (29.2)	97 (70.8)	1		1		
Water only	98 (55.1)	80 (44.9)	2.9 (1.8,4.7)	0.001	2.5 (0.85, 7.5)		0.09
Presence of latrine facility							
Yes	133 (43.5)	173 (56.5)	1				
No	5 (55.6)	4 (44.4)	1.6 (0.43,6.17)	0.47			
Hand washing purpose after latrine (with)							
Soap and water	11 (19.6)	45 (80.4)	1		1		
Water only	24 (51)	23 (49)	4.3 (1.7, 10.2)	0.001*	5.36 (1.78,16)		0.003*
Access to health education							
Yes	31 (41.3)	44 (58.7)	1				
No	107 (44.6)	133 (55.4)	1.14 (0.7, 1.9)	0.62			

*Clinical significance of the association

^a^Crude odds ratio

^b^Adjusted odds ratio

^c^Average monthly income in USD (for year 2019)

As multivariate analysis showed lack of handwashing habit after latrine, being a farmer and walking barefoot were significant predictors of IPI where the pregnant women lacking habit of handwashing after latrine, being a farmer and walking barefoot were 5.36, 7.3 and 6.13 times more often infected with intestinal parasitosis than their counterparts ([AOR, 95% CI 5.36 (1.78, 16), P = 0.003], [AOR, 95% CI 7.3 (1.46, 37.00), P = 0.03], [AOR, 95% CI 6.13 (1.98, 19.00), P = 0.002]), respectively (Table 3).

## Discussion

Ethiopia is a developing country where IPIs are major public health problems with the high prevalence reported [6, 7]. The burden of intestinal parasites, particularly the soil-transmitted helminths (STHs), is often very high in school children and pregnant women [3, 14]. High prevalence of hookworm was reported in some parts of Ethiopia like East Wollega districts [4], Gilgel Gibe dam area [7] and Anbesame health center [8]; whereas northern and southern part of Ethiopia in Wondo Genet [5] and Mecha districts [6] were infected with high dominance of *Ascaris lumbricoides*.

A cross-sectional study conducted in similar study settings like in Colombia, Venezuela, Nigeria, Gabon, and Kenya revealed 1.2%, 73.9%, 56.8%, 49% and 25.23% of IPI respectively [10, 16–19]. Intestinal parasitosis is one of the most prevalent infectious diseases in the tropical and subtropical areas of the world. It is a medical and public health problem in sub-Saharan countries including Ethiopia. Pregnant women are one of the most vulnerable groups for this infection due to their immune suppression during their pregnancy [15].

According to WHO, IPI is considered a public health problem if the prevalence of IPI is greater than 20% [1]. Accordingly, with the prevalence of 43.8%, intestinal parasitosis is one of the major public health concerns in the study area. This finding was comparable with findings from Nigeria 43.8% [20] and Gilgel Gibe dam area, South West Ethiopia 41% [7] and Gabon 49% [18], but was lower than the findings from Venezuela 73.9% [10], Makurdi, Benue state 56.8% [17], and Mecha district, North West Ethiopia (70.6%) [5]; however, our finding was higher when compared to findings reported from Bogota, Colombia (1.2%) [16], Nepal 35% [21], Kwale district, Kenya 25.23% [19], Bahir Dar, North West Ethiopia 31.5% [11], Gandhi Memorial Hospital, Addis Ababa 25.2% [22] and Debre Markos, North West Ethiopia 27.4% [23].

These variations could be attributable to smaller sample size, the difference in the geographical area and cultural practices, difference in implementation of various intervention strategies, the difference in study settings and time of the study, and the difference in location of the subjects under consideration. The specific type of study subjects, the methods employed for stool examination, and the time of study may have also contributed to the variation [10, 16, 17, 19].

The most identified parasite in the study area was hookworm 106 (33.7%) followed by *Ascaris lumbricoides* 23 (7.3%) which was in agreement with reports from other areas in Ethiopia [4, 7, 8]. But, different reports from Venezuela [10], Nepal (21), Kenya [24] and Wondo Genet, Southern Ethiopia [5] indicated the commonest parasite was Ascaris lumbricoides. This disparity might be due to the difference in geography, wearing shoes and level of income.

In our study, being a farmer, walking barefooted and absence of appropriate handwashing habit after latrine significantly increases intestinal parasitic infection. This finding was comparable with the study conducted in Kenya [24] and Mecha district, South Ethiopia [5], but it was not consistent with the study conducted in Gandhi Memorial Hospital, Addis Ababa [22]. This might be attributed to the difference in residence, socio-demography, and level of awareness.

In this study, pregnant women with a habit of walking barefoot were six times more likely infected by hookworm than who wear shoe regularly. This finding was comparable with the study conducted in Mecha district, Hosanna, South Ethiopia and Anbesame, North West Ethiopia were not wearing shoes regularly increases the odds of infection higher than their counterparts. This is because the larvae of hookworm penetrate exposed human skin from contaminated soil [5, 8, 25].

Those pregnant women from farmer households were more likely infected by intestinal parasites when compared with a government employee. This finding was comparable with the study done in Ibadan of Nigeria [20] and Kitale district, Kenya [24] where being farmers (engaged in agricultural activity) had statistically significant association with IPI since this activity enables them to have frequent contact with contaminated soil.

In the present study, washing hands with water only after latrine increases the odds of IPI in pregnant women by 5.36 folds higher. This finding was consistent with the study done in Makurdi, Nigeria [17], Anbesame and Bahir Dar, North West Ethiopia [8, 11] whereas it was inconsistent with the study done in East Wollega [4] and Hosanna, Southern Ethiopia [25].

Unlike our result, research done in Hosanna, Southern Ethiopia reported unprotected sources of water, family size, and low monthly income (< 35 USD) had a positive association with IPI [25].

### Conclusion

The prevalence of IPI was significantly high in the study area where pregnant mothers were mostly affected by hookworm infection and ascariasis. The determinant factors were being a farmer, walking barefooted and absence of handwashing habit after latrine. To alleviate this burden, intervention like periodic treatment (deworming), health education, improving sanitation and awareness creation on shoe wearing habit for pregnant women should be given.

## Limitation of the study

Limitations of our study include the use of the small sample size and a single stool specimen to assess infection status, which may have underestimated the worm burden. Also, we recommend further study on the anemic status of the participants.

## Data Availability

All data generated or analyzed during this study are included in this manuscript and in case needed it can be accessible up on formal request to the corresponding author.

## Abbreviations

ANC: antenatal care
AOR: adjusted odds ratio
COR: crude odds ratio
ETB: Ethiopian Birr
HC: Health Center
HH: households
IPI: intestinal parasitic infection
IQC: internal quality control
RPM: revolution per minute
SOP: standard operating procedure
STH: soil transmitted helminthes
WHO: World Health Organization
